# Thyroid Hormones and Metabolic Syndrome: A Narrative Review of Findings from 18 Years of Follow-up in Tehran Thyroid Study (TTS)

**DOI:** 10.5812/ijem-168079

**Published:** 2026-02-28

**Authors:** Atieh Amouzegar, Mohamadamin Tarighat-Payma, Safdar Masoumi, Alireza Amirabadizadeh, Hengameh Abdi, Fereidoun Azizi, Ladan Mehran

**Affiliations:** 1 Endocrine Research Center, Research Institute for Endocrine Disorders, Research Institute for Endocrine Sciences, Shahid Beheshti University of Medical Sciences, Tehran, Iran; 2 Medical Toxicology and Drug Abuse Research Center (MTDRC), Birjand University of Medical Sciences, Birjand, Iran

**Keywords:** Metabolic Syndrome, Thyroid Hormones, Hypothyroidism, Hyperthyroidism

## Abstract

**Context:**

Thyroid dysfunction and metabolic syndrome (MetS) share similar pathophysiological features, suggesting a bidirectional relationship between these two prevalent endocrinological disorders. This review summarizes findings from 18 years of follow-up in the Tehran Thyroid Study (TTS), a large community-based prospective cohort, focusing on the association between thyroid function and MetS.

**Evidence Acquisition:**

We systematically reviewed TTS publications (until 2025) on thyroid function and MetS. Exposures included thyroid-stimulating hormone (TSH), free thyroxine (FT4), thyroid peroxidase antibody (TPOAb), and thyroid feedback indices [Thyroid Feedback Quantile-Based Index (TFQI), Thyrotroph T4 Resistance Index (TT4RI), and TSH Index (TSHI)]. Outcomes were MetS and its components. Study designs ranged from cross-sectional and prospective cohort to joint longitudinal/time-to-event models.

**Results:**

Cross-sectional TTS analyses showed an inverse association between FT4 (but not TSH) and the prevalence of MetS. Among 3,755 euthyroid adults, a 1-unit increase in FT4 was associated with lower odds of MetS (OR 0.96, 95% CI: 0.92 - 0.99). Across thyroid functional states, overt hypothyroidism had the highest MetS prevalence (41.6%); in men, overt hypothyroidism was associated with higher odds of MetS versus euthyroidism (OR 2.9, 95% CI: 1.04 - 8.40). Prospective TTS analyses supported a modest thyroid-to-MetS direction. In a 10-year cohort of 2,393 participants without MetS at baseline, declining FT4 over follow-up predicted incident MetS in non-obese adults (OR 0.57, 95% CI: 0.34 - 0.96) and was associated with lower odds of incident abdominal obesity and hypertriglyceridemia. In joint longitudinal-time-to-event models (n = 1,436; median 9-year follow-up), higher time-varying TSH was associated with higher MetS risk (HR 1.17, 95% CI: 1.01 - 1.28), while higher FT4 predicted lower MetS risk (HR 0.54, 95% CI: 0.29 - 0.97). In the reverse direction, baseline MetS did not independently predict incident thyroid dysfunction over 9 years (n = 4,905; adjusted HR, 0.95; 95% CI: 0.77 - 1.18). Thyroid hormone sensitivity indices were not associated with MetS overall, although higher TFQI was associated with hypertension (OR 1.14, 95% CI: 1.05 - 1.23).

**Conclusions:**

Eighteen years of follow-up in TTS suggest a modest, mostly one-way thyroid-MetS association. Higher (or high-normal) TSH and lower FT4 modestly increase MetS risk, whereas baseline MetS did not significantly increase the risk of later thyroid dysfunction.

## 1. Context

Metabolic syndrome (MetS) is a cluster of central obesity, insulin resistance, dyslipidemia, and hypertension, which has a significant contribution to the high global burden of cardiovascular disease, diabetes, and mortality (1). In Iran, MetS prevalence is approximately 31% (95% CI: 28 - 34%) and is higher in females and older age groups (2). A systematic review and meta-analysis also estimated the incidence of MetS in the Iranian general population to be about 98 per 1,000 person-years (2020 pooled estimate), underscoring its dynamic public health impact (3).

Thyroid dysfunctions are among the major endocrine diseases following diabetes, affecting 10% to 18% of the adult population worldwide. In the United States and European countries, subclinical and overt hypothyroidism affect 4 - 5% and 0.3% of adults, respectively (4). In Iran, recent data indicated comparable or higher prevalence of 2 - 3% for overt hypothyroidism, 5 - 6% for subclinical hypothyroidism, and 1 - 3% for hyperthyroidism (5). Thyroid dysfunction exerts widespread adverse metabolic effects. Hypothyroidism tends to increase weight, LDL, and total cholesterol and blood pressure, while hyperthyroidism often induces insulin resistance and lipid abnormalities even in subclinical forms (6). Both thyroid disease and MetS independently predispose to metabolic abnormalities and cardiovascular events as they share similar underlying features such as visceral adiposity, dyslipidemia, and insulin resistance (6-8).

Given the similar pathophysiological pathway, it has been hypothesized that there is a bidirectional association between thyroid hormones and MetS (8, 9). Higher rates of thyroid autoimmunity and hypothyroid phenotypes have been observed among the MetS population based on cross-sectional and longitudinal reports (9). On the other hand, recent meta-analyses indicated the association of subclinical hypothyroidism with central obesity, elevated triglycerides, low HDL, and hypertension (9, 10). The development of subclinical hypothyroidism in euthyroid individuals having MetS has been reported inconsistently (11, 12).

Despite these suggestive observations, important gaps remain. The majority of prior studies are cross-sectional or of short duration, limiting causal inference about temporal order. Conventional analyses often rely on a single baseline thyroid measurement, overlooking the dynamic nature of thyroid hormone regulation. Furthermore, most epidemiological work has solely focused on thyroid-stimulating hormone (TSH) and free thyroxine (FT4) levels, and composite indices of thyroid feedback sensitivity (such as TSH index, FT3/FT4 ratio, or thyroid feedback quantile-based index) have seldom been applied. Emerging data suggest that such indices may provide additional insight; for example, higher TSH-based resistance indices and lower FT3/FT4 ratios have been correlated with greater MetS severity (13), but these metrics remain rarely used. In short, standardized definitions and prospective designs are needed to clarify causality and mechanism.

The Tehran thyroid study (TTS) helps address these needs. Tehran thyroid study is a long-standing, community-based prospective cohort established in 1999 - 2001 as part of the Tehran Lipid and Glucose Study. It enrolled 5,786 adult residents (aged ≥ 20) and has performed triennial examinations over nearly two decades (14, 15). Serial measurements of TSH, FT4, thyroid antibodies, and cardiometabolic parameters enable longitudinal analysis of the interplay between thyroid and MetS. In particular, TTS data have been analyzed with joint modeling approaches that combine repeated hormone measurements with time-to-event outcomes (16). These methods capture the evolving relationship between thyroid hormones and incident MetS more accurately than static models. In this review, we integrate 18-year findings from TTS with international literature, highlighting how prolonged follow-up clarifies the bidirectional associations of thyroid status and MetS.

## 2. Evidence Acquisition

We conducted a comprehensive search for publications from the TTS group addressing thyroid function and MetS. Following the approach of prior TTS reviews, we searched PubMed, Scopus, Web of Science, and the library of the Research Institute for Endocrine Sciences for articles. We included peer-reviewed TTS studies (original cohort, longitudinal, or cross-sectional analyses) that evaluated thyroid-related exposures and metabolic outcomes. Exposures were thyroid function indices (TSH, FT4, TPOAb) and derived indices of thyroid hormone sensitivity (thyroid feedback quantile-based index [TFQI], thyrotroph T4 resistance index [TT4RI], and TSH index [TSHI]). Outcomes included MetS and its individual components (waist circumference, triglycerides, HDL cholesterol, fasting plasma glucose, and blood pressure). Extracted data included study design (cross-sectional, prospective cohort, joint modeling), sample size and characteristics, follow-up duration, thyroid exposures, metabolic outcomes, and statistical methods. TTS studies used a variety of methods, including simple cross-sectional comparisons, generalized estimating equations (GEE) for repeated measures, multivariable logistic or linear regression, and advanced joint longitudinal/time-to-event models. Table 1 provides a summary of the included studies and their principal findings.

**Table 1.** Summary of Published Papers on Thyroid Hormones and Metabolic Syndrome in the Framework of Tehran Thyroid Study

Study	Design/Analysis	N	Population (Inclusion)	Follow-up	Thyroid Exposures	Outcomes (MetS, etc.)	Main Findings (OR/HR [95% CI], P-Value)	Covariates
**Mehran et al. 2014 (** 17 **)**	Cross-sectional (TTS baseline); multivariable logistic/linear regression	3755	Euthyroid adults ≥ 20 yr (no known thyroid disease, diabetes, or steroid/lipid meds)	Cross-sectional	FT4, TSH	MetS and components (waist, TG, HDL, BP, glucose)	Higher FT4 concentrations, even within the euthyroid range, were independently associated with a lower prevalence of MetS). The prevalence of MetS decreased progressively across increasing FT4 tertiles (from 30.1% in the lowest tertile to 22.4% in the highest; P < 0.001). Each one-unit increase in FT4 was associated with a 4% reduction in the odds of MetS (OR = 0.96; 95% CI: 0.92 - 0.99). Serum TSH showed no independent association with MetS after multivariable adjustment.	Age, sex, smoking, BMI, HOMA-IR
**Mehran et al. 2017 (** 18 **)**	Prospective cohort (TTS); multivariable logistic regression for incident outcomes	2393 (free of MetS at baseline)	Adults ≥ 20 yr without MetS at baseline; excluded overt thyroid disease, thyroid meds, BMI < 18.5	10 y	Change in FT4 (baseline → follow-up); Baseline FT4	Incident MetS and components (abdominal obesity, TG, BP, glucose)	An increase in FT4 levels over the follow-up period was associated with a substantially lower risk of developing abdominal obesity (OR = 0.49; 95% CI: 0.35 - 0.69) and hypertriglyceridemia (OR = 0.57; 95% CI: 0.41 - 0.78). Conversely, higher FT4 levels were associated with an increased risk of incident hypertension (OR = 1.35; 95% CI: 1.05 - 1.74). Baseline FT4 was inversely associated with incident MetS (OR = 0.59; 95% CI: 0.39 - 0.90); however, this association was attenuated and lost statistical significance after adjustment for BMI.	Age, sex, smoking, BMI, HOMA-IR
**Mehran et al. 2017 (** 19 **)**	Cross-sectional (TTS baseline); Logistic regression	5422	Adults ≥ 20 yr (excluded thyroid disorders, pregnancy, severe illness)	Cross-sectional	Thyroid function status (euthyroid vs. subclinical/overt hypo-/hyperthyroidism)	MetS prevalence and components	The prevalence of MetS was highest among participants with overt hypothyroidism (41.6%). In men with overt hypothyroidism, the odds of MetS were nearly threefold higher compared with euthyroid men (OR = 2.9; 95% CI: 1.04 - 8.40). Subclinical hypothyroidism was associated with a higher prevalence of MetS only among individuals older than 50 years. Both overt and subclinical hyperthyroidism were associated with increased odds of hyperglycemia after full multivariable adjustment.	Age, sex, BMI, smoking
**Mehran et al. 2021 (** 12 **)**	Prospective cohort (TTS); Cox regression / time-to-event analysis	4905	Adults ≥ 20 yr (euthyroid at baseline for some analyses); Excluded those on steroids/RAI/pregnancy where noted	9 y	Baseline MetS (assessed as exposure); TSH/FT4 measured for outcome analyses	Incident thyroid dysfunction (subclinical/overt hypo- or hyperthyroidism; TPOAb positivity)	Baseline MetS was not independently associated with the subsequent development of thyroid dysfunction during follow-up. After multivariable adjustment, the hazard ratio for any incident thyroid disorder among participants with MetS compared with those without MetS was 0.95 (95% CI: 0.77 - 1.18). The incidence rates of thyroid dysfunction were similar in individuals with and without MetS (13.9 vs. 15.0 per 1,000 person-years, respectively).	Age, sex, BMI, smoking, TPOAb positivity
**Mehran et al. 2022 (** 20 **)**	Cross-sectional (TTS survey 2008-2011); Multivariable logistic regression	5124 (euthyroid sample analyzed)	Tehran adults ≥ 20 y (euthyroid sample, from 2008 - 2011 survey)	Cross-sectional	Thyroid hormone sensitivity indices: TFQI, PTFQI, TSHI, TT4RI	Diabetes, hypertension, MetS components	Higher TFQI, reflecting reduced central sensitivity to thyroid hormones, was significantly associated with a higher prevalence of diabetes mellitus (OR = 1.16; 95% CI: 1.04 - 1.30; P = 0.009) and elevated blood pressure (OR = 1.14; 95% CI: 1.06 - 1.23). In addition, higher TSHI (OR = 1.22; 95% CI: 1.08 - 1.38) and TT4RI (OR = 1.08; 95% CI: 1.01 - 1.16) were independently associated with hypertension, but not with diabetes.	Age, sex, smoking, physical activity, BMI, HOMA-IR
**Amirabadizadeh et al. 2025 (** 16 **)**	Prospective cohort (TTS); Joint longitudinal-time-to-event model	1436	Adults ≥ 20 yr (TTS participants with repeated measures)	Median = 9 y (per manuscript)	Longitudinal TSH and FT4 trajectories (time-varying)	Incident MetS and components (time-to-event)	In joint longitudinal-time-to-event models, each one-unit increase in log-transformed TSH over time was associated with a 17% higher risk of incident MetS (HR = 1.17). In contrast, each one-unit increase in FT4 was associated with a 46% lower risk of developing MetS (HR = 0.54). Higher log-TSH trajectories were also associated with an increased risk of hypertension, whereas higher FT4 trajectories were linked to reduced risks of abdominal obesity and hypertriglyceridemia.	Age, sex, education, physical activity, BMI, HOMA-IR, etc.
**Abiri et al. 2023 (** 21 **)**	Cross-sectional (ANCOVA)	2988	Euthyroid adults	Cross-sectional	FT4, TSH	Obesity phenotypes (MHNW, MHO, MUO)	Mean FT4 concentrations were significantly higher in metabolically healthy normal-weight individuals compared with those with metabolically healthy obesity or metabolically unhealthy obesity (P < 0.001). No significant differences in TSH levels were observed across obesity phenotypes (P = 0.260). The observed differences in FT4 were significant only among participants younger than 55 years.	Age, waist circumference, smoking, physical activity
**Amouzegar et al. 2018 (** 14 **)**	Narrative review/TTS overview (20 yr follow-up)	-	TTS cohort overview	20 y (TTS)	Serial FT4, TSH measures	IR, MetS, BP, CVD (summary)	Across approximately two decades of follow-up in the TTS, higher FT4 levels were consistently inversely associated with insulin resistance, and declining FT4 over time predicted the development of MetS. FT4 showed a positive association with blood pressure, while TSH was linked to systolic and diastolic blood pressure, particularly in men. No consistent associations between thyroid function and cardiovascular disease outcomes were observed.	As reported in original studies (age, sex, BMI)
**Mehran et al. 2019 (** 8 **)**	Narrative review	-	-	-	-	-	Evidence from the TTS indicates heterogeneous and context-dependent associations between normal-range thyroid hormone levels and MetS. These relationships appear to be modified by age, sex, BMI, insulin resistance, and smoking status. While low-normal thyroid function may influence individual metabolic parameters, its role in determining overall MetS risk remains inconclusive.	-

Abbreviations: TTS, Tehran thyroid study; FT4, free thyroxine; TPOAb, thyroid peroxidase antibody; TFQI, Thyroid Feedback Quantile-Based Index; PTFQI, Parametric Thyroid Feedback Quantile-Based Index; TSHI, Thyroid-Stimulating Hormone Index; TT4RI, Thyrotroph T4 Resistance Index.

### 2.1. Definitions

MetS was defined by Joint Interim Statement in all studies (22). Central sensitivity to thyroid hormones refers to the responsiveness of the hypothalamic-pituitary axis to the negative feedback effects of thyroid hormones, particularly T4 and T3. Central sensitivity describes the extent to which a given level of circulating free thyroid hormones (FT4 or FT3) suppresses or stimulates TSH secretion. In epidemiological and clinical studies, central sensitivity is assessed using composite indices, including: Thyrotroph T4 Resistance Index (TT4RI), TSH Index (TSHI), and Parametric Thyroid Feedback Quantile-Based Index (PTFQI) (20, 23). TPOAb positivity was defined as a TPOAb >35 IU/mL in women and TPOAb > 32.8 IU/mL in men. The TSH reference range was defined as 0.32 - 5.06 mU/L. Euthyroidism was defined as the absence of thyroid dysfunction, including both clinical and subclinical forms of hypothyroidism and hyperthyroidism, using the reference ranges of TSH (0.32 - 5.06 mU/L) and FT4 (0.91 - 1.55 ng/dL) derived from the current population (24). Insulin resistance assessed by the homeostasis model assessment of insulin resistance (HOMA-IR) is calculated as: HOMA-IR = [Fasting insulin [µIU/mL] × Fasting glucose (mmol/L)] / 22.5 (25).

## 3. Results

### 3.1. Effect of Thyroid Hormones on Metabolic Syndrome

The effect of thyroid hormones on MetS has been investigated in TTS using various methodologies. A cross-sectional study examined the link between thyroid hormones and MetS in 3,755 euthyroid subjects (17). The findings highlighted that FT4, rather than TSH, was associated with MetS and its components in this group. The prevalence of MetS was found to decrease across FT4 tertiles, from 30.1% in the lowest to 22.4% in the highest (P < 0.001). Correspondingly, higher serum FT4 levels were associated with lower odds of having MetS (OR: 0.96, 95% CI: 0.92 - 0.99). In contrast, serum TSH showed no significant association with MetS, although it was positively correlated with high triglycerides (P = 0.04) and insulin resistance (P < 0.001); the link to triglycerides, however, disappeared after adjusting for the homeostasis model assessment of insulin resistance (HOMA-IR) (P = 0.17).

A large cross-sectional analysis within the TTS involving 5,422 participants investigated the prevalence of MetS across different thyroid function statuses (19). The study found that individuals with overt hypothyroidism had the highest prevalence of MetS at 41.6%, as well as the highest rates of abdominal obesity (45%) and hypertriglyceridemia (58%) compared to other groups (P < 0.05). After adjusting for age and smoking, a significant association between overt hypothyroidism and MetS was observed, but only in men, who had 2.9-fold increased odds of the syndrome (OR: 2.9, 95% CI: 1.04 - 8.4). While subclinical hypothyroidism was not linked to MetS in the general population, a subgroup analysis revealed that individuals over 50 with this condition had a significantly higher risk (OR: 1.76, 95% CI: 1.04 - 2.97). Conversely, both overt and subclinical hyperthyroidism were associated with significantly higher odds of hyperglycemia, with the highest prevalence seen in subclinical hyperthyroidism (31.3%, P < 0.001).

To assess the impact of hormonal changes over time, a 10-year prospective cohort study analyzed data from 2,393 TTS participants who were free of MetS at baseline (18). This study uniquely evaluated how longitudinal variations in thyroid hormones predicted the incidence of MetS. The findings indicated that a decrease in serum FT4 values over the follow-up period was associated with an increased risk for developing MetS, particularly in non-obese adults (BMI < 30 kg/m²). While the association between changing FT4 levels and MetS lost statistical significance after adjusting for BMI in the overall population, it remained a significant predictor in the non-obese subgroup, even after full adjustment (OR = 0.57, 95% CI: 0.34 - 0.96). An increase in FT4 over time was also associated with lower odds of developing abdominal obesity (OR = 0.49, 95% CI: 0.35 - 0.69) and hypertriglyceridemia (OR = 0.57, 95% CI: 0.41 - 0.78). Consistent with other TTS findings, variations in TSH levels over time were not associated with the incidence of MetS or any of its components.

A more recent longitudinal investigation within the TTS, involving 1,436 participants over a median follow-up of 9 years, employed a novel joint modeling technique to simultaneously analyze hormone trajectories and the time to MetS incidence (16). This advanced statistical approach revealed that higher longitudinal TSH levels were significantly associated with an increased risk of developing MetS. After adjusting for factors including BMI and HOMA-IR, each one-unit increase in the log-transformed TSH level corresponded to a 17% higher risk of incident MetS (HR: 1.17, 95% CI: 1.01 - 1.28). Higher TSH was also specifically linked to a greater risk of hypertension (HR: 1.08, 95% CI: 1.02 - 1.22). Conversely, higher longitudinal FT4 levels were associated with a 46% reduced risk of MetS (HR: 0.54, 95% CI: 0.29 - 0.97) and a lower risk of abdominal obesity (P = 0.04) and hypertriglyceridemia (P = 0.01). However, a sex-stratified analysis presented a contradictory finding, suggesting that higher FT4 levels were significantly associated with an increased risk of MetS in both men and women.

### 3.2. Thyroid Hormone Sensitivity and Metabolic Syndrome

In a cross-sectional study, the association between reduced sensitivity to thyroid hormone and MetS was investigated in 5,124 participants, with the primary analysis focusing on 4,319 euthyroid individuals (20). The findings indicated that there was no significant association between thyroid hormone sensitivity and MetS as a complete entity for any of the indices, with fully adjusted model P-values of 0.9 for TFQI, 0.5 for TSHI, and 0.7 for TT4RI. However, the study found significant associations with individual components of MetS. A 1-SD increase in TFQI, indicating reduced thyroid hormone sensitivity, was associated with higher odds of having high blood pressure (OR = 1.14, 95% CI: 1.05 - 1.23) but was associated with lower odds of having a high waist circumference (OR = 0.93, 95% CI: 0.86 - 0.99) and high LDL cholesterol (OR = 0.91, 95% CI: 0.84 - 0.99). Similarly, a 1-SD increase in both TSHI and TT4RI was associated only with higher odds of having high blood pressure (TSHI: OR = 1.08, 95% CI: 1.01 - 1.15; TT4RI: OR = 1.08, 95% CI: 1.01 - 1.16), with no significant associations with any other MetS components observed for these two indices in the euthyroid group.

### 3.3. Effect of Metabolic Syndrome on Thyroid Function

In a prospective cohort study, the impact of MetS on thyroid function and the incidence of thyroid disorders was investigated over approximately 9 years of follow-up (12). The study included 4,905 participants, of whom 32% had MetS at baseline. The initial cross-sectional analysis showed that the prevalence of overt hypothyroidism was significantly higher in the MetS group compared to the non-MetS group (1.4% vs. 0.7%, P = 0.02). However, this association disappeared after adjusting for key confounding factors such as age, sex, BMI, smoking, and TPOAb positivity (OR=1.14, 95% CI: 0.81 - 1.62). The longitudinal analysis further demonstrated that the incidence rate of developing any thyroid dysfunction was 13.9 per 1,000 person-years in the MetS group compared to 15.0 in the non-MetS group (P = 0.46). The multivariate-adjusted HR for developing any thyroid dysfunction in subjects with MetS was 0.95 (95% CI: 0.77 - 1.18). More specifically, the adjusted HRs for subclinical hypothyroidism, overt hypothyroidism, and TPOAb positivity were 0.96 (95% CI: 0.74 - 1.25), 0.86 (95% CI: 0.52 - 1.42), and 0.90 (95% CI: 0.65 - 1.25), respectively. This study concluded that when important confounders are adequately considered, MetS is not independently associated with thyroid hypofunction or an increased incidence of thyroid disorders. In another study, metabolically healthy normal weight participants had higher levels of FT4 when compared with metabolically healthy or unhealthy obese subjects (P < 0.001), even after adjustment for the confounding variables. No difference was observed in the levels of TSH among obesity phenotypes. They concluded that metabolic abnormality was independently related to low normal FT4 levels in overweight/obese euthyroid individuals (21).

### 3.4. Bidirectional Interplay Between Thyroid Function and Metabolic Syndrome

The pathophysiologic mechanisms of thyroid dysfunction and MetS are closely linked (8). Thyroid hormones regulate basal metabolic rate, lipid and glucose metabolism, and vascular tone. Therefore, hypothyroidism (even subclinical) leads to weight gain, hyperlipidemia, and insulin resistance (26). In turn, signals from metabolism and inflammation can influence the hypothalamic-pituitary-thyroid axis. Leptin and cytokines produced by adipose tissue stimulate TRH and TSH secretion. Insulin resistance may also alter the peripheral conversion of T4 to T3. Several studies describe this as a bidirectional relationship. Increased adiposity raises TSH levels and can blunt thyroid function. Thyroid status, in turn, affects adiposity and insulin sensitivity (8, 26).

In practice, some studies report that obesity and MetS features correlate with higher TSH (even in the euthyroid range) and thyroid autoantibodies (27, 28). However, in TTS, the prospective analysis showed that MetS did not significantly alter thyroid hormone trends or incident thyroid disease after accounting for confounders (12). These findings suggest that overt MetS does not always lead to thyroid hypofunction and subtle changes in the thyroid axis, such as a gradual rise in TSH within the normal range, may still occur (29).

The TTS findings largely align with many international cohorts linking low thyroid activity to MetS, but there are notable discrepancies (30, 31). Consistent with TTS, several cross-sectional studies have found higher normal-range TSH or lower FT4 associated with MetS components (29, 32). For example, a Chinese cohort reported that impaired central thyroid sensitivity (higher TFQI, TSHI, TT4RI) was significantly associated with prevalent MetS (ORs up to 1.57 per SD increase) and greater MetS severity (13). These findings mirror the TTS trend that a hypothyroid shift favors MetS development. By contrast, some studies report no independent thyroid-MetS link after adjusting for BMI or use different thyroid parameters (33). Overall, TTS results are mostly consistent with the notion that even high-normal TSH or lower FT4 may flag elevated metabolic risk (20). Discrepancies may arise from variations in confounder adjustment, ethnicity, iodine status, or MetS definitions. Importantly, most studies emphasize that normal thyroid tests do not always reflect complete thyroid metabolic health. Multiple studies show that even thyroid hormone and TSH values classified as normal can mask underlying subtle thyroid dysfunction that affects metabolic parameters such as lipid profiles, blood pressure, glucose metabolism, and overall cardiovascular risk.

## 4. Conclusions

### 4.1. TTS Findings on Thyroid Function and Metabolic Syndrome

Recent TTS analyses highlight a clear link between thyroid status and MetS (14, 17). In longitudinal TTS data, higher TSH levels over time were associated with increased MetS incidence, while higher FT4 was protective. Specifically, over a 9 - 10-year follow-up, each SD increase in log‐TSH raised MetS risk (HR = 1.17) and each SD increase in FT4 cut risk (HR = 0.54) (16). Correspondingly, TTS investigators reported that declining FT4 predicted future MetS in euthyroid/subclinical patients (17, 18). In cross-sectional analyses, FT4 inversely correlated with insulin resistance, suggesting low-normal thyroid function promotes MetS features (17). In contrast, baseline comparisons of MetS vs non-MetS groups in TTS showed no independent differences in TSH or FT4 after adjustment (12). Nevertheless, MetS participants did have more overt and subclinical hypothyroidism at unadjusted baseline, which disappeared when adjusted for age, BMI, and other factors (12). TTS also explored novel thyroid-sensitivity metrics. One report found that thyroid hormone resistance (TFQI) was significantly associated with diabetes and high blood pressure in euthyroid subjects, but no association was found with MetS (20). Most international studies reported that reduced thyroid hormone sensitivity is linked to a higher risk of MetS (13, 34, 35). These mixed TTS findings suggest complex, possibly stage-dependent relationships between thyroid feedback and metabolism (36).

### 4.2. Clinical Implications

These findings indicate that clinicians should stay alert for thyroid abnormalities in patients with MetS or its components. While universal thyroid screening for MetS is not usual, TTS data suggest that even high-normal TSH or decreasing FT4 levels might help identify individuals at greater risk for cardiometabolic issues. It could be wise to regularly monitor thyroid function in MetS patients, especially if they develop hypertension, dyslipidemia, or glucose intolerance. Conversely, the presence of thyroid dysfunction, including subclinical hypothyroidism, should prompt assessment of MetS features. Additionally, emerging thyroid sensitivity indices may eventually improve risk stratification, as indicated by both TTS and international cohorts. However, further evidence is necessary before these indices can be implemented clinically. Meanwhile, controlling traditional MetS risk factors remains critical, with a low threshold to check thyroid tests if there is worsening metabolic control.

### 4.3. Limitations of TTS Data

The TTS findings come from an observational cohort, so causality cannot be confirmed. Potential confounding factors, such as BMI, lifestyle, iodine intake, or autoimmunity, may affect both thyroid hormone levels and MetS risk despite multivariate adjustments. Although TTS is large and measured over a long period, participant dropout over 18 years and changes in assay methods could bias the results. The use of thyroid sensitivity indices, while innovative, depends on statistical measures whose normative values and clinical significance are still developing. Lastly, TTS is specific to certain ethnicities and regions; the findings may not apply to other populations with different genetics or iodine levels.

### 4.4. Future Directions

To address remaining uncertainties, future research should involve larger longitudinal studies and interventional trials. Advanced joint-modeling approaches illustrate methods to link time-varying thyroid levels with MetS risk; similar methods could be used across different groups. Validating thyroid sensitivity indices in other ethnicities and through prospective designs would help clarify their predictive value. Mechanistic studies (such as on deiodinase activity, thyroid receptors, or reverse-T3 levels in MetS) could elucidate causal pathways. Genetic research might identify variants that affect both thyroid function and metabolic outcomes. Lastly, clinical trials involving modest thyroid hormone adjustments (such as treating high-normal TSH) could determine if altering thyroid status positively influences MetS progression.

## Data Availability

The dataset presented in the study is available on request from the corresponding author during submission or after publication.
